# Techniques Used for Analyzing Microplastics, Antimicrobial Resistance and Microbial Community Composition: A Mini-Review

**DOI:** 10.3389/fmicb.2021.603967

**Published:** 2021-03-26

**Authors:** Simona Bartkova, Anne Kahru, Margit Heinlaan, Ott Scheler

**Affiliations:** ^1^Department of Chemistry and Biotechnology, Tallinn University of Technology, Tallinn, Estonia; ^2^Laboratory of Environmental Toxicology, National Institute of Chemical Physics and Biophysics, Tallinn, Estonia; ^3^Estonian Academy of Sciences, Tallinn, Estonia

**Keywords:** antimicrobial resistance, microplastics, heavy metals, plastisphere, emerging technologies, antibiotics

## Abstract

Antimicrobial resistance (AMR) is a global health threat. Antibiotics, heavy metals, and microplastics are environmental pollutants that together potentially have a positive synergetic effect on the development, persistence, transport, and ecology of antibiotic resistant bacteria in the environment. To evaluate this, a wide array of experimental methods would be needed to quantify the occurrence of antibiotics, heavy metals, and microplastics as well as associated microbial communities in the natural environment. In this mini-review, we outline the current technologies used to characterize microplastics based ecosystems termed “plastisphere” and their AMR promoting elements (antibiotics, heavy metals, and microbial inhabitants) and highlight emerging technologies that could be useful for systems-level investigations of AMR in the plastisphere.

## Introduction

The increasing resistance of pathogenic bacteria to common antibiotics (AB) found in human and veterinary settings worldwide (WHO, 2018) highlights the urgent need for improved surveillance programs (Dadgostar, 2019) and research to hinder further escalation of antimicrobial resistance (AMR) (Interagency Coordination Group on Antimicrobial Resistance, 2019). Although the number is debatable (de Kraker et al., 2016), according to O’Neill (2016), the global annual death toll due to AMR could rise to 10 million by 2050.

The emerging contaminant—plastic—has potential to further enhance AMR by providing porous micro ecosystems termed “plastisphere” (Keswani et al., 2016). In the environment, plastic does not biodegrade but fragmentizes into smaller fractions such as microplastics (MPs) (1 μm–5 mm) (Frias and Nash, 2019) or further into nanoplastics (NPs) (≤1 μm) (Gigault et al., 2018). MPs have been increasingly detected in all the ecosystems, though due to rapid microbial colonization and subsequent density changes, about 70% of the MPs in the aquatic environment sedimentates and thus the sediments, along with soils that receive MPs contamination from sludge application, have been considered as the sinks of MPs (Nizzetto et al., 2016; Corradini et al., 2019; Schmiedgruber et al., 2019). Plastic is also ingested and inhaled by humans (Cox et al., 2019; de Wit and Bigaud, 2019) as indicated by detection of plastic in stool samples (Schwabl et al., 2019) and human lung tissue (Pauly et al., 1998), respectively. Compared to MPs, NPs have been scarcely studied due to limitations of current analytical techniques (Nguyen B. et al., 2019), yet Besseling et al. (2019) have speculated future NP concentrations in mass may become 10^14^ times higher than currently measured MP concentrations.

**FIGURE 1:**
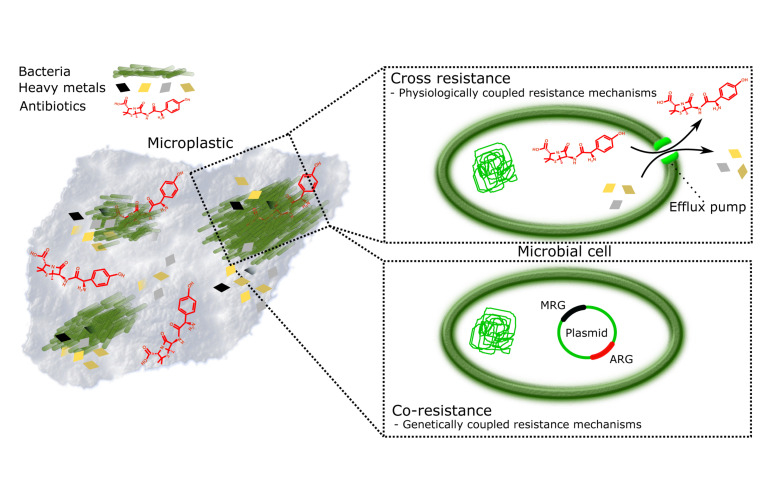
Plastisphere is a potential hotspot for evolution of antimicrobial resistance (AMR). Persistence and surface characteristics of microplastics make it an excellent reservoir of microbes and pollutants, such as heavy metals (HMs) and antibiotics (ABs). Together they form miniature ecosystems, plastispheres, where AMR is promoted by (i) cross-resistance where resistance mechanisms to HMs and ABs are physiologically coupled, for example, efflux pumps (Baker-Austin et al., 2006; Seiler and Berendonk, 2012); and (ii) co-resistance where antibiotic resistance genes (ARGs) and metal resistance genes (MRGs) are present on the same mobile genetic element and thus genetically coupled, whereby selection for metal resistance in, for example, animal gut, and anthropogenically contaminated soils and water bodies lead to automatic selection of ARGs (Baker-Austin et al., 2006; Seiler and Berendonk, 2012; Li et al., 2017).

The plastisphere creates a habitat that promotes attachment of and subsequent biofilm production by microbes (Zettler et al., 2013). In this habitat, the microbes are also in close vicinity of MP-associated pollutants, such as (ABs) and heavy metals (HMs) (Figure 1). This combination of being surrounded by pollutants while being protected by biofilm can lead to possible change in the microbial species distribution (Munier and Bendell, 2018; Imran et al., 2019). ABs are considered to be the primary drivers of AMR (Kraemer et al., 2019), originating largely from inefficient wastewater treatment processes and pharmaceutical discharge (Wilkinson and Boxall, 2019). HMs are accumulating in the environment *via* waste flows from industrial activities (mining, smelting, fertilizer use, sewage sludge application), but may also be mobilized due to natural processes (e.g., bedrock weathering) (Ali et al., 2019; Zhou et al., 2020). HM pollution drives the selection for metal resistance genes (MRGs) and correlates with increased occurrences and amount of antibiotic resistance genes (ARGs) (Figure 1; Baker-Austin et al., 2006; Li et al., 2017; Nguyen C.C. et al., 2019).

It is hypothesized that weathering can intensify both HM (Prunier et al., 2019) and AB association (Zhou et al., 2020) with MPs and potential migration of additives (Commission Regulation, 2011) from the polymer. Indeed, the European Chemical Agency has identified 1,550 additives (European Chemical Agency, 2019), many of which are known to leach into the environment (De-la-Torre et al., 2020; Bolívar-Subirats et al., 2021) as they are generally not chemically bound to polymers and can thus potentially migrate (Hahladakis et al., 2018). Metals and metal-based additives are mostly used as colorants and fillers, and research on release rate of toxic HM additives (e.g., Cd, Pb, Sb, Sn) in plastics during recycling is ongoing (Hahladakis et al., 2018). Long-term impact of MP pollution on the development of AMR is yet unclear, but AB retention, ARG presence, and exchange of ARGs through horizontal gene transfer among bacteria on MPs has been shown (Imran et al., 2019; Yang et al., 2019; Zhu et al., 2019).

In this mini-review, we discuss the role of plastisphere in the development of AMR, and the current technologies used to address various aspects of AB-HM-MP pollution and highlight the data gaps, novel techniques, and approaches.

## Characterization of Plastisphere-Associated Antibiotics and Heavy Metals

**FIGURE 2:**
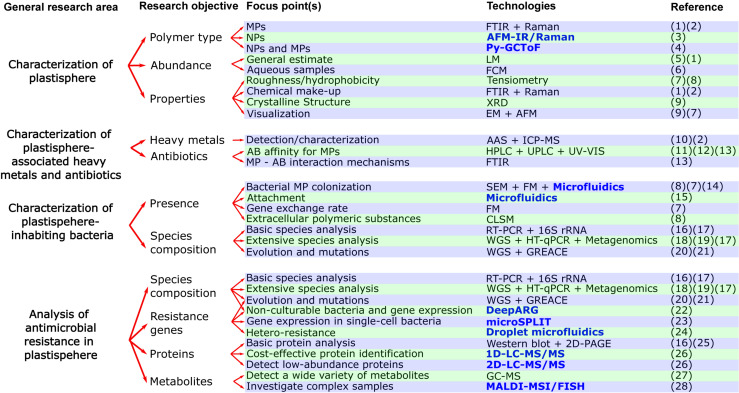
Flow chart with current and novel (written in bold and blue color) approaches for studying the effects of plastisphere-associated pollutants (heavy metals, antibiotics) on antimicrobial resistance. Abbreviations for technologies in alphabetical order: AAS, Atomic absorption spectroscopy; AFM, Atomic Force Microscopy; AFM-IR/Raman, Atomic force microscopy infrared/Raman; CLSM, Confocal Laser Scanning Microscopy; DeepARG, Deep learning model for antibiotic resistance genes; EM, Electron microscopy; FCM, Flow cytometry; PFGE, Pulsed-field gel electrophoresis; FM, Fluorescence microscopy; FTIR, Fourier transform infrared microscopy; GC-MS, Gas chromatography–mass spectrometry; GREACE, Genome Replication Engineering Assisted Continuous Evolution; HPLC, High-performance liquid chromatography; HT-qPCR, High-throughput qPCR; ICP-MS, Inductively coupled plasma mass spectrometry; LM, Light microscopy; MALDI-MSI/FISH, Matrix assisted laser desorption/ionization–Mass spectrometry imaging/Fluorescence *in situ* hybridization; microSPLIT, Microbial Split-Pool Ligation Transcriptomics; Py-GCToF, Pyrolysis–Gas Chromatography Time of Flight Mass Spectrometry; RT-PCR, Reverse transcription polymerase chain reaction; SEM, Scanning Electron Microscopy; UPLC, Ultra-performance liquid chromatography; UV-VIS, Ultraviolet–visible spectrophotometry; XRD, X-ray diffraction; WGS, Whole genome sequencing; 1D/2D-LC-MS/MS, One dimensional/Two dimensional online separation-liquid chromatography-tandem mass spectrometry; 2D-PAGE, Two-dimensional gel electrophoresis. References in numerical order: (1) = (Zhang Y. et al., 2020), (2) = (Imhof et al., 2016), (3) = (Fu et al., 2020), (4) = (Sullivan et al., 2020), (5) = (Gimiliani et al., 2020), (6) = (Kaile et al., 2020), (7) = (Dussud et al., 2018), (8) = (Hossain et al., 2019, (9) = (Li et al., 2018), (10) = (Munier and Bendell, 2018), (11) = (Bolívar-Subirats et al., 2021), (12) = (Zhang et al., 2018), (13) = (Yu et al., 2020b), (14) = (Pousti et al., 2019), (15) = (Secchi et al., 2020), (16) = (Leng et al., 2019), (17) = (Meier et al., 2020), (18) = (Pathak et al., 2020), (19) = (Zhao Y. et al., 2019), (20) = (Li X. et al., 2019), (21) = (Qin et al., 2019), (22) = (Cuadrat et al., 2020), (23) = (Kuchina et al., 2020), (24) = (Scheler et al., 2020), (25) = (Bar et al., 2007), (26) = (Hinzke et al., 2019), (27) = (Li W. et al., 2019), (28) = (Geier et al., 2020).

MP abundance and polymer type are determined by microscopy and spectroscopy methods (Figure 2). The first steps(s) in analyzing plastic from environmental samples usually comprises of different separation and/or purification procedures. Separation frequently consists of passing samples through sieves or filter membranes (Fu et al., 2020). The purification process commonly involves treatment with, for example, ethanol (Zettler et al., 2013; Dussud et al., 2018), purified sea water (Dussud et al., 2018), or strong acidic and/or alkaline solutions (Cole et al., 2014; Imhof et al., 2016). Stereomicroscopes are used for the general estimate of MPs in environmental samples but also to characterize their surface, size, and shape (Gimiliani et al., 2020; Zhang Y. et al., 2020). Roughness and hydrophobicity of MPs is evaluated by tensiometry, measuring the contact angle of water drops (Dussud et al., 2018; Hossain et al., 2019). For visualization with a greater resolving power, scanning electron microscopy (SEM) (Arias-Andres et al., 2018; Li et al., 2018), or atomic force microscopy (AFM) (Dussud et al., 2018) are used. The main difference between stereomicroscopy and SEM is their resolution limit of around 200 and 2 nm, respectively. AFM has a third dimension of magnification (the *z*-axis), enabling constructing landscape maps of surfaces. Spectroscopy methods based on molecular vibration such as Raman spectroscopy (Zettler et al., 2013; Amaral-Zettler et al., 2015; Imhof et al., 2016), Fourier Transform Infrared Spectroscopy (FTIR)(Bryant et al., 2016; Laganà et al., 2019; Zhang Y. et al., 2020), and attenuated total reflection-FTIR (Amaral-Zettler et al., 2015; Viršek et al., 2017) allow to decipher MPs chemical makeup for more precise identification. X-ray diffraction can provide the crystalline structure of MPs (Li et al., 2018). Nevertheless, there is a gap in research, because current technologies still have difficulties in accurately detecting and characterizing the chemical properties of extensively degraded plastics (especially MPs ≤50 μm and NPs) from complex environmental samples (Lehner et al., 2019). Examples include lacking a standard procedure for separating and/or purifying samples from different matrices and using purification steps that may damage the plastic (Lö et al., 2017), microscopy techniques not providing information on plastic composition (Müller et al., 2020), and spectroscopy techniques like Raman and FTIR not having the resolution power needed for NP characterization (Imhof et al., 2016; Mason et al., 2018; Fu et al., 2020).

Absorption of light and mass-to-charge ratio are used to measure the content of HMs within and on the surface of MPs *via* atomic absorption spectroscopy (Brennecke et al., 2016; Cabral et al., 2016; Munier and Bendell, 2018) and inductively coupled plasma mass spectrometry (ICP-MS) (Rochman et al., 2014; Cabral et al., 2016; Imhof et al., 2016), respectively (Figure 2). The latter being the gold standard for detecting and characterizing metals. AB affinity for MP has been studied in batch adsorption experiments in laboratory settings. High-performance liquid chromatography (HPLC) coupled to a diode array detector (Guo et al., 2019; Guo and Wang, 2019) and to a triple quadruple detector (Bolívar-Subirats et al., 2021) and ultra-performance liquid chromatography coupled to a photodiode array detector (Zhang et al., 2018) but also UV-visible spectroscopy have been used to determine MP-sorbed AB (Wan et al., 2019; Yu et al., 2020a,b; Figure 2). In addition, FTIR has been used to characterize the interaction mechanisms between MPs and ABs (Wan et al., 2019; Yu et al., 2020a,b; Figure 2). Although technology has allowed in-depth analysis of HMs and ABs, there is a knowledge gap on how the affinity for pollutants differs from MPs and NPs degraded from larger plastic due to weathering processes to primary MPs and NPs.

## Characterization of Plastisphere-Inhabiting Bacteria

MPs in water bodies form an ideal substratum for bacterial biofilm formation as they adsorb nutrients and organic matter from the essentially nutrient-poor water habitat supporting the growth of bacteria. Generally, the colonization of MPs is a very rapid process (within 24 h) depending on a variety of factors (Oberbeckmann et al., 2015) of which environmental factors and not the plastic type have recently been shown to be the most significant influencer for microbial composition on MPs (Wright et al., 2020).

Bacterial association with MPs is analyzed by SEM and fluorescence microscopy) (Zettler et al., 2013; Bryant et al., 2016; Arias-Andres et al., 2018; Dussud et al., 2018; Hossain et al., 2019; Figure 2). Fluorescence can further examine the gene exchange rate within biofilm communities and planktonic bacteria (Arias-Andres et al., 2018), accomplished *via* fluorescent self-transmissible plasmids (Arias-Andres et al., 2018). To study the extracellular polymeric substances of the biofilm matrix, confocal laser scanning microscopy (CLSM) is used (Hossain et al., 2019; Figure 2). This is due to CLSM’s ability of obtaining high-resolution images in various depths of a sample, usually 50–100 μm in biological samples (Jonkman et al., 2020). Overall, research in this area has greatly expanded due to the above-mentioned technologies; however, there is a gap in analysis and modeling of microbial colonization of both MPs and NPs in different environmental settings. Change in species diversity is mostly investigated by sequencing variable regions from the conserved 16S ribosomal RNA (16S rRNA) (Knapp et al., 2017; Zhao et al., 2018; Zhao Y. et al., 2019; Chen et al., 2019; Learman et al., 2019; Meier et al., 2020; Figure 2). Sequencing methods, including whole genome sequencing (WGS), are also ideal for studying effect of HMs on resistance-related genes in bacteria (Pathak et al., 2020; Figure 2).

Metagenomics with possible combination of metatranscriptomics permits analysis of the species present in the microbial community, including non-culturable bacteria, while simultaneously studying regulation of ARGs, MRGs, and other genes at the mRNA/functional level within the whole community (Cabral et al., 2016; Meier et al., 2020; Figure 2). Functional metagenomics is useful for screening of resistance genes that are expressed in specific environments such as HM polluted sites, while further allowing discovery of possible genes with novel functions (Cheng et al., 2012; Staley et al., 2015). Further methods for routine examination are polymerase chain reaction (PCR) techniques (Medardus et al., 2014; Knapp et al., 2017; Chen et al., 2019), although here, only a limited number of genes are investigated (Zhang Y. et al., 2020). This can be overcome by high-throughput qPCR chip technologies (Zhao et al., 2018; Zhao Y. et al., 2019) and WGS (Learman et al., 2019; Figure 2). Alternations in gene expression levels caused by HM exposure can be determined by reverse transcription PCR for specific genes (Leng et al., 2019) or with metatranscriptomics for the whole transcriptome (Cabral et al., 2016). Many different ecosystems have now been investigated for ARGs and MRGs, yet a comprehensive overview of the ARG and MRG prevalence remains to be done. Another gap is single-cell bacterial research, as the effect of HMs and plastic on AMR at the level of single-bacterium is virtually non-existent. This is mainly due to lack of technologies being able to extract and analyze their genetic material (Kuchina et al., 2020).

Sequencing in combination with long-term experiments can detect mutations that occur in bacteria during prolonged growth in HM rich environments (Chi et al., 2017; Li X. et al., 2019; Qin et al., 2019). These experiments include serial long-term culturing of resistant mutants exposed to subtoxic levels of HMs, followed by WGS (Li X. et al., 2019). Genome Replication Engineering Assisted Continuous Evolution is another alternative, in which evolution of resistant mutants is accelerated before sequencing (Qin et al., 2019; Figure 2). *In vivo* experiments with mice being exposed to HMs *via* oral administration followed by sequencing of the gut microbiota have shed light on the effect of HMs in mammals *in vivo* (Chi et al., 2017). There are several ways to explore how HMs can have an effect on the protein and metabolite level in bacterial monocultures. A frequently used method for proteomics is two-dimensional gel electrophoresis (Bar et al., 2007; Figure 2). Another way to explore protein expression is through liquid chromatography-tandem mass spectrometry (LC-MS/MS) where proteins are first separated by LC and then ionized and characterized by mass-to-charge ratio and relative abundance (Li W. et al., 2019; Figure 2). Western blotting is another widely applied technique (Leng et al., 2019). For studying metabolites, gas chromatography-mass spectrometry is usually performed (Leng et al., 2019; Figure 2). Although working with monoculture bacteria does not mimic the true situation of microbial interaction in the environment, possible impact of HMs and plastics on the proteomics/metabolomics level in different types of bacteria is currently still a research gap in need of investigation. This gap needs to be addressed in the light of recent studies, such as the one by Li W. et al. (2019), showing that an alternation in bacterial metabolic pathways may affect their AMR. Exposure to HMs has already shown the ability to alter metabolic pathways of bacteria in the gut (Chi et al., 2017); nonetheless, there is still much to be learned on possible influence of HMs and perhaps plastics on the different pathways and the interplay with AMR.

## Overcoming the Challenges to the Plastisphere Characterization Research Gaps

The most optimal solution for future development of a standard method for quantifying and characterizing the composition of smaller fractions of MPs, including NPs (<1 μm), might be to merge completely new analytical methods with the existing technologies (Nguyen B. et al., 2019; Fu et al., 2020). For now, combining AFM with infrared spectroscopy or Raman seems promising, since AFM offers relatively simple sample preparation, and samples can be conserved during analysis (Fu et al., 2020; Figure 2). One disadvantage is, however, that obtaining quality imaging of the sample depends on how flat and smooth the sample is (Fu et al., 2020), making it difficult for simultaneous investigation of NPs and larger MPs. To obtain a more complete overview, at least in aqueous samples, we recommend analysis of plastics on PTFE membranes combined with Pyrolysis-Gas Chromatography Time of Flight Mass Spectrometry (Py-GCToF) (Sullivan et al., 2020; Figure 2). This analytical method is based on analyzing thermal degradation products, and it has shown to be fast, reliable, and have high resolution (Sullivan et al., 2020). A second option for aqueous samples that might be more easily standardized for future environmental identification and quantification of MPs and NPs (0.2–2 μm), is flow cytometry in combination with staining and cell sorting (Kaile et al., 2020).

Change in MPs and NPs composition and their affinity for pollutants and microbes can be uncovered by merging analytical and sequencing technology with *in situ* and *ex situ* experiments. *Ex situ* batch sorption experiments provide the opportunity to focus on specific parameters (Li et al., 2018; Zhang H. et al., 2020), while *in situ* studies are necessary to observe the real-life complex interaction of MPs and NPs with their surroundings (Oberbeckmann et al., 2017). Depending on the environment investigated, suitable analytical techniques for detection and characterization of MPs and NPs in such experiments could be either Py-GCToF (Sullivan et al., 2020) or micro-FTIR and Raman spectroscopy (Lö et al., 2017; Figure 2). HPLC and ICP-MS could further be used for AB and HM detection, respectively (Cabral et al., 2016; Zhang H. et al., 2020; Figure 2). Finally, microbial analysis in the experiments would need to include both analytical tools for studying biofilm (e.g., CLSM) and metagenomic sequencing for discovering possible ARGs and MRGs as well as species diversity (Cabral et al., 2016; Hossain et al., 2019).

Accumulation of MPs in the food chain and the effect on spread of AMR should be investigated by long-term *in vivo* studies combined with multidisciplinary tools such as NGS sequencing, ICP-MS, and vibrational spectroscopy methods. Previous *in vivo* studies focusing on influence of MP accumulation are inconsistent in their methods and yield conflicting results (Van Raamsdonk et al., 2020). One issue with standardizing *in vivo* studies is the complexity of the sample material, making it difficult to detect MPs. This could be solved by an enzymatic purification method for MPs/NPs developed by Lö et al. (2017), which can remove organic and inorganic material from different matrices while not affecting the polymers and couple it to micro-FTIR and Raman spectroscopy (Figure 2). Lö et al. (2017) provides a step-by-step guide to the enzymatic purification, which includes optional subdivision of samples, usage of specific buffers, and lipase and amylase for samples with high lipid or polysaccharide content.

## Novel Approaches and Methods for Addressing AMR Knowledge Gaps in the Plastisphere

Obtaining a wider overview of microbial communities, including spread of ARGs and MRGs in different habitats, is feasible with modern NGS approaches. There are two aspects that should be considered in future analyses: (1) presence of non-culturable bacteria and (2) expression level of resistant genes in the bacterial communities. Integrating metagenomics and metatranscriptomics with machine-learning tools such as DeepARG, trained to find the existing and novel ARGs and MRGs, is a suitable option for this challenge (Arango-Argoty et al., 2018; Cuadrat et al., 2020; Figure 2). Studying heterogeneous modulation of gene expression by HMs (and MPs/NPs) in a single bacterium is possible, but single-cell RNA sequencing (scRNA-seq) studies are still scarce due to differences from eukaryotic cells such as low mRNA content and lack of polyadenylation. This challenge could be overcome by the scRNA-seq platform Microbial Split-Pool Ligation Transcriptomics (Kuchina et al., 2020; Figure 2). The approach was recently adapted for *Bacillus subtilis* and *Escherichia coli* by Kuchina et al. (2020) and has advantages such as: (1) no need for single cell physical isolation, (2) compatibility with a wide range of cell shapes and sizes; and (3) enables use of un-encapsulated and fixed cells (Ma et al., 2019).

Proteomic and metabolomic pathways in bacteria play an important role in AMR (Li W. et al., 2019). 1D and 2D-LC-MS/MS spectrometry and mass spectrometry imaging (MSI) can be used for analyzing the impact that MPs and HMs potentially have on proteomics/metabolomics activity levels in the bacteria. In their extensive testing of LC-MS/MS spectrometry, Hinzke et al. (2019) suggest the most cost-effective method for maximizing the number of identified proteins by MS is online separation by 1D-LC. For a more precise guidance for specific objectives, we refer to the flow chart and overall work of Hinzke et al. (2019) (Figure 2). Though, regardless of technology used, it is essential that proteome bioinformatics progresses in parallel with the recent advances in MS methods; otherwise, proteomic analysis will remain limited (Ameen and Raza, 2017; Petriz and Franco, 2017). In congruence, technology needed for analysis of metabolites is restricted as well (Dunham et al., 2017). One promising method is MSI, because it provides chemical and spatial analysis and methods can easily be adapted to specific environmental samples. MSI works by distinguishing chemical compounds *via* their mass-to-charge ratio, and currently, there are three MSI methods commercially available for analyzing bacteria (Dunham et al., 2017). Though the main limiting factor is that any single MSI experiment only gives a fraction of the metabolites present in samples (Dunham et al., 2017). Nevertheless, we believe that when combined with other technologies, it could pave the way for future metabolomic research as exemplified by the work of Geier et al. (2020), where matrix-assisted laser desorption/ionization MSI was combined with FISH microscopy (Figure 2). This enabled linking metabolomes to groups of 50–100 microbial cells in complex environmental samples and be able to resolve single-cell bacteria in the near future (Geier et al., 2020).

Microbial colonization and ability to form biofilm are also heterogenous characteristics of microbes, and they play a key role in AMR. Microfluidic platforms show great potential for enabling complex biofilm studies (Yawata et al., 2016; Pousti et al., 2019), including the scarcely researched effect of flow rate and motility of bacteria on attachment (Secchi et al., 2020; Figure 2). Bacteria communities, both hetero and isogenic, can contain cells with diverse range of resistance (El-Halfawy and Valvano, 2015). Droplet microfluidic technology could be the most promising tool for such investigation because it allows high-throughput culturing of bacteria at wide range of isolated conditions (Kaminski et al., 2016; Scheler et al., 2020; Figure 2).

## Conclusion

In this mini-review, we highlighted technologies that have been used for analyzing different aspects of plastisphere-associated AMR. Although we found that many different aspects of AMR have been explored through multiple studies using advanced methods, knowledge gaps remain. To address these gaps, we summarize currently available technologies potentially suitable for future research. This should provide analytical tools for scientists of diverse backgrounds seeking answers for complex urgent problems: HM- and AB-contaminated plastisphere-associated promotion of AMR.

## Author Contributions

All authors listed have made a substantial, direct and intellectual contribution to the work, and approved it for publication.

## Conflict of Interest

The authors declare that the research was conducted in the absence of any commercial or financial relationships that could be construed as a potential conflict of interest.
